# Reply to the commentary entitled “Environmental estrogens have an impact on the ERs and their signaling in ER responsive tissues and organs” by Xu-Guang Guo

**DOI:** 10.1007/s11255-016-1269-0

**Published:** 2016-03-29

**Authors:** Peng Zhang, Wan-Li Hu, Bei Cheng, Xing-Huan Wang

**Affiliations:** Department of Urology, Zhongnan Hospital, Wuhan University, 169 Donghu Load, Wuchang District, Wuhan, 430071 Hubei People’s Republic of China; Department of Anatomy and Embryology, Wuhan University School of Basic Medical Sciences, Wuhan University, Wuhan, 430071 Hubei People’s Republic of China

Editor,

We appreciate the comments of Xu-Guang Guo on our manuscript entitled: Which play a more important role in the development of large-sized prostates (≥80 ml), androgen receptors or oestrogen receptors? A comparative study.

Various factors might contribute to the bias of our research results. Not only the possible differences on the patients’ occupation or living-habit (patients might be exposed to different environmental estrogens) leading to various degrees of endocrine disruptors but also other unknown factors could do it. These relationships discussed in epidemiologic studies were more properly due to their large sample. Our study was designed as a pilot study with small sample. Because all the enrolled patients were from the single-center hospital and the quite small sample, it was hardly to find a statistical difference on the mentioned confounding factors among the three groups.

In our study, it was a great hypothesis that low levels of circulating estrogens might indirectly cause enlargement of the prostate, by reducing the inhibition of pituitary gonadotropin secretion, leading to increased testicular output of androgens and elevated circulating androgen levels which was not just testosterone. Furthermore, it might well be elevated that the plasma dihydrotestosterone (DHT) levels in the large-sized prostatic patients although it was not measured in our study. The testosterone could be converted into DHT by the action of 5α-reductase isozymes in peripheral tissues such as prostate and skin. Whether increased plasma testosterone levels being converted into DHT in the large-sized prostatic patients resulted in the circulating testosterone levels reverted was needed to explore in the future [1]. Anyway, in our study the expression of androgen receptors in the large-sized prostate was significantly increasing, and this suggested that ARs more or less had an important role in the development of large-sized prostate.

We agreed that the tissue weight was also a good method to determine the degree of the prostatic hyperplasia, but the study was designed as a prospective study and it was needed to know the prostate volume before the surgery. It was more convenient for us to gain the information on the patients’ prostatic volume by transrectal ultrasounds (TRUS).

Although the International Prostate Symptom Score (IPSS) did not significantly differ between the groups, but it was demonstrated that men with growing prostates were at a greater risk of symptomatic deterioration. An agent against the prostate growth must be a potential for the management of BPH. How will patients feel when their large-sized prostates became a small-sized prostate [2]? We very expected that time will come.
